# Dissociable sensitivity and bias mechanisms mediate behavioral effects of exogenous attention

**DOI:** 10.1038/s41598-019-42759-w

**Published:** 2019-09-02

**Authors:** Vishak Sagar, Ranit Sengupta, Devarajan Sridharan

**Affiliations:** Centre for Neuroscience, Indian Institute of Science, C. V. Raman Avenue, Bangalore, 560012 India

**Keywords:** Attention, Attention, Human behaviour, Human behaviour

## Abstract

Attention can be directed endogenously, based on task-relevant goals, or captured exogenously, by salient stimuli. While recent studies have shown that endogenous attention can facilitate behavior through dissociable sensitivity (sensory) and choice bias (decisional) mechanisms, it is unknown if exogenous attention also operates through dissociable sensitivity and bias mechanisms. We tested human participants on a multialternative change detection task with exogenous attention cues, which preceded or followed change events in close temporal proximity. Analyzing participants’ behavior with a multidimensional signal detection model revealed clear dissociations between exogenous cueing effects on sensitivity and bias. While sensitivity was, overall, lower at the cued location compared to other locations, bias was highest at the cued location. With an appropriately designed post-cue control condition, we discovered that the attentional effect of exogenous pre-cueing was to enhance sensitivity proximal to the cue. In contrast, exogenous attention enhanced bias even for distal stimuli in the cued hemifield. Reaction time effects of exogenous cueing could be parsimoniously explained with a diffusion-decision model, in which drift rate was determined by independent contributions from sensitivity and bias at each location. The results suggest a mechanistic schema of how exogenous attention engages dissociable sensitivity and bias mechanisms to shape behavior.

## Introduction

Selection of behaviorally relevant information can occur either by endogenous (top-down) engagement of attention, based on task-relevant goals (e.g. monitoring a traffic light), or by exogenous (bottom-up) capture of attention, by salient sensory events (e.g. a flash of lightning)^1–4^. There exists an extensive literature on the behavioral effects of endogenous visuospatial attention^5–7^. In contrast, exogenous visuospatial attention’s effects have been more challenging to study, perhaps because exogenous capture of attention is automatic and transitory, operating on fast timescales of a few tens to hundreds of milliseconds^8,9^. Previous studies have reported systematic effects of exogenous cueing of attention on behavioral accuracy^10–15^ as well as reaction times at the cued location^16–19^. Yet, it is unclear if these effects of cueing reflect the operation of a unitary attentional process, or of multiple underlying processes.

**Figure 1:**
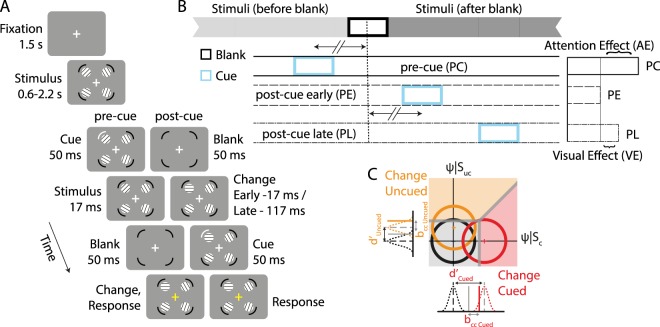
Multialternative change detection task with exogenous cueing. (**A**) Change detection task to measure the effects of exogenous cueing of attention, with pre-cue (PC), early post-cue (PE) and late post-cue (PL) trials (see text for details). (**B**) Timing of exogenous cues for the different trial types. (Top) Light grey bar: stimuli presented before the blank. Dark grey bar: stimuli presented after the blank. (Left) Schematics showing the timing of exogenous cue (blue, 50 ms duration) relative to the midpoint of the blank epoch (dashed vertical line), about which information relevant to detecting the change occurred. In PC and PE trials the midpoint of cue presentation occurred symmetrically about (67 ms before or after) the midpoint of the blank (horizontal arrows with two slashes). In PL trials the mid-point of cue presentation occurred 167 ms after the midpoint of the blank. The exogenous cue is expected to produce both attentional and visual effects in PC trials, visual effects, but no attentional effects, in PE trials, and possibly weak visual effects in PL trials. (Right) Attention effects (AE) of the cue are computed by subtracting behavioral metrics in PE trials from corresponding metrics in PC trials, whereas visual effects (VE) of the cue are computed by subtracting metrics in PL trials from corresponding metrics in PE trials. (**C**) Schematic of a two-dimensional m-ADC signal detection model. Decision variables for each location are represented along orthogonal decision axes in a multidimensional decision space. Circles: Contours of multivariate Gaussian decision variable distributions. Black: noise distribution; red and orange: signal distribution for “change” trials at the cued and uncued locations, respectively. Thick gray lines: Decision surfaces for the multialternative decision. Gray, red and orange shading: Decision zones corresponding to no-change, change at cued location and change at uncued location, respectively. Univariate Gaussians along the axes represent the marginal decision variable distributions at each location.

It is increasingly clear that attention, when cued endogenously, facilitates behavior through at least one of two mechanisms: i) by enhancing the sensory processing of attended information, often at the cost of unattended information, and ii) by prioritized gating of attended information for guiding perceptual decisions^20–24^. These component mechanisms have been recently studied in the context of endogenous attention tasks^25–30^ using signal detection theory (SDT), a highly successful framework for the analysis of behavior^31,32^. Conventional SDT specifies distinct sensory and decisional parameters for quantifying attention’s effects on perceptual decisions –sensitivity (d′) and choice criterion (or bias, b_cc_; Fig. 1C), respectively. While a change in sensitivity reflects the improvement in signal-to-noise for the attended information, a change in bias reflects prioritized gating of attended information for decisions^33–35^.

Here, we study the effect of exogenous, visuospatial attention on sensitivity and bias. Does exogenous cueing of attention affect sensitivity, bias, or both? Are these sensitivity and bias effects of exogenous cueing coupled or dissociable? Can the sensitivity and bias effects be mechanistically linked to accuracy and reaction time effects? Do exogenous cueing effects differ from corresponding effects of endogenous cueing of attention^29^?

To answer these questions, we designed a multialternative change detection task with exogenous spatial cueing (Fig. 1A) and analyzed the data with a recently developed multidimensional signal detection model (the m-ADC model)^28–30^. Analysis with the m-ADC model revealed striking dissociations between sensitivity and bias changes produced by exogenous cueing. Combining the m-ADC model with a drift-diffusion model^36^, we develop a mechanistic framework for understanding how exogenous cueing improves behavioral accuracies and reaction times through systematic effects on sensitivity and bias.

## Results

### A multialternative task design for quantifying attentional effects of exogenous cueing

We investigated the effects of exogenous cueing of attention on perceptual processing and decision-making. Specifically, we investigated how exogenous cueing modulates sensitivity and choice bias at cued and uncued locations, and whether these effects were coupled or dissociable.

Subjects (n = 45) performed a multialternative change detection task with exogenous cueing (see Methods for details). Each trial began with a display of 4 stimulus gratings with different, randomized orientations, one in each visual quadrant. After an unpredictable delay, an exogenous cue (high contrast arc) appeared briefly (50 ms) adjacent to one of the four stimuli (Fig. 1A), with equal probability across locations (25%). 17 ms following the offset of the cue, the screen was blanked for 50 ms, after which the gratings reappeared. Upon reappearance, any one of the four gratings, or none, had changed in orientation; the change could occur at any location independently of the cued location, rendering the cue’s spatial location irrelevant to the task. At the end of each trial, subjects reported the location of orientation change, or no change, with one of five distinct button presses (Supplementary Fig. S1).

To account for sensory interference by the exogenous cue (“visual interference” effects), we designed two additional, post-cueing control conditions (Fig. 1A,B): (i) “early” post-cue (PE) trials in which the exogenous cue was presented17 ms after the stimuli reappeared, and (ii) the “late” post-cue (PL) trials in which the exogenous cue was presented 117 ms after the stimuli reappeared. The PE trial was designed so that the pre-cue and post-cue were symmetrically distributed about a time point coincident with the centre of the blank epoch; note that information relevant to localizing the change (grating orientation before versus after the change) occurred symmetrically, on either side of the blank epoch (Fig. 1B). The PL trial was designed so that the post-cue occurred more than 100 ms after the change event. Similar post-cueing task designs, which control for the net visual stimulation provided by the cue, have been employed in previous studies^37–40^.

**Figure 2:**
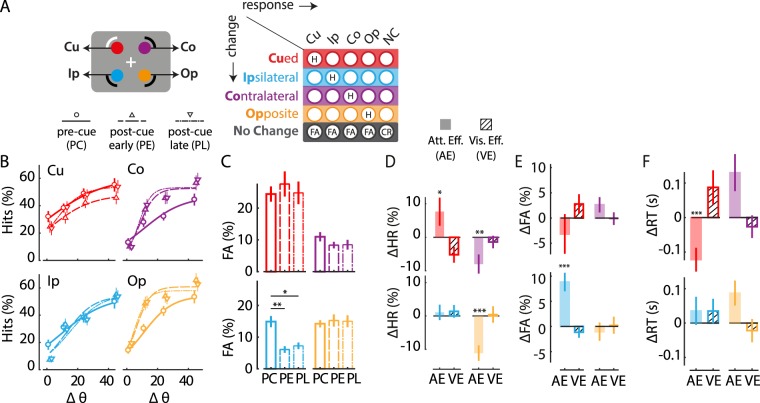
Effects of exogenous cueing on hit rates, false alarm rates, and reaction times. (**A**) (Top) Schematic representation of the four locations relative to the exogenously cued location. Red: cued location (Cu); cyan: cue-ipsilateral location (Ip); purple: cue-contralateral location (Co); orange: cue-opposite location (Op). (Bottom) 5 × 5 stimulus-response contingency table in the 4-ADC task. Rows represent locations of change and columns represent locations of response, both measured relative to the cue. Color conventions are as in the location schematic. Gray: No-change (NC) trials. H: Hit, FA: False Alarm, CR: Correct Rejection. (**B**) Psychometric function (data pooled across n = 45 subjects) showing hit rates for different magnitudes of grating orientation change (Δθ) for Cu (upper left panel), Ip (lower left panel), Co (upper right panel) and Op (lower right panel). Circles and solid lines: Data and sigmoid fit for pre-cue (PC) trials. Upright triangles and dashed lines: Data and sigmoid fit for early post-cue (PE) trials. Inverted triangles and dash-dot lines: Data and sigmoid fit for late post-cue (PL) trials. Color conventions are as in panel (A). Error-bars: s.e.m. (**C**) False-alarm rates for the four locations (Cu, Ip, Co, Op) and three trial types (PC, PE, PL). Other conventions are as in panel (B). (**D**) Modulation of hit-rates (averaged across orientation change values) by exogenous cueing for the four locations (Cu, Ip, Co, Op). Color conventions are as in panel (C). Filled bars: Attention effect (AE = m_PC_ − m_PE_). Hatched bars: Visual effect (VE = m_PE_ − m_PL_). Asterisks: significance levels (*p < 0.05, **p < 0.01, ***p < 0.001). Other conventions are as in panel (B). (**E**) Same as in panel D, but for false-alarm rates. Other conventions are as in panel (D). (**F**) Same as in panel D, but for reaction times. Other conventions are as in panel (D).

Each of these trial types occurred in a pseudorandom sequence in equal proportions (1/3rd each) across trials. For each trial type (PC, PE, PL), subjects’ responses were aggregated into a 5 × 5 stimulus-response contingency table (Fig. 2A, Supplementary Fig. S1), and analyzed with the m-ADC model to yield sensitivity and bias at each location. In Fig. 1C we show a schematic of a two-dimensional (2-ADC) model, for illustrative purposes, although these data were analyzed with a four-dimensional (4-ADC) model (see Methods).

We then quantified the attentional and visual interference effects of exogenous cueing. Subtracting each behavioral metric (e.g. sensitivity or bias) in the PE trials from its value in the PC trials yielded the attentional effects of exogenous cueing, at each location (AE(m) = m_PC_ − m_PE_; Fig. 1B). On the other hand, subtracting the corresponding behavioral metrics across the PL and PE trials yielded the visual interference effects of exogenous cueing (VE(m) = m_PE_ − m_PL_; Fig. 1B; for details, see Methods section on *“Computing attention and visual interference effects*.*”*). In results reported subsequently, we use the following terminology: when we report metrics averaged across locations in the quadrants contralateral to the cue (Co and Op locations) we term these Co_av_. When we report metrics averaged across all uncued locations (Ip, Co, and Op), we term these Uc_av_.

### Exogenous cueing produces mixed effects on accuracies and reaction times

First, we quantified exogenous attention’s effects on psychometric functions of hit rates (Fig. 2B), false-alarm rates (Fig. 2C), and correct rejection rates (Supplementary Fig. S1). Each metric revealed distinct patterns of benefits and costs across locations and cue conditions. On average, both hit rates and false-alarm rates were highest at the cued location (PC, HR: p = 0.016; FA: p < 0.001), and not significantly different across uncued locations. Cueing produced opposite patterns of modulations of hit rates at the cued (Cu) and two uncued locations in the cue-contralateral hemifield (Co, Op). While cueing increased hit rates significantly at the cued location in PC, as compared to PE trials (Cu: AE(hits) = 7.7+/−4.2%; mean +/− standard error, p < 0.013; Fig. 2D), it decreased hit rates significantly at the cue-contralateral uncued (Co and Op) locations (Co_av_: AE(hits) = −10+/−2.1%, p < 0.001). On the other hand, cueing did not produce significant changes in false alarm rates at any of these locations (Cu: AE(false-alarm) = −3.2+/−3.9%, p = 0.748; Co_av_: AE(false-alarm) = 0.8+/−1.2%, p = 0.365; Fig. 2E). A notable exception to all of these trends was the cue-ipsilateral (Ip) location. In contrast to the other uncued locations, cueing did not significantly modulate hit rates at the Ip location (Ip: AE(hits) = 1+/−2.3%, p = 0.354). On the other hand, it produced a striking increase in false alarm rates in PC trials (Ip: AE(false-alarm) = 8.8 +/−1.8%, p < 0.001; Fig. 2E). In addition, overall correct rejection rates were lower in PC trials as compared to PE trials (AE(correct-rejection) = −7.3+/−4.6%, p = 0.046; Supplementary Fig. S1).

Exogenous cueing produced similar, mixed patterns of effects on reaction times (RT), across cued and uncued locations. Cueing effects on reaction times closely matched the effects on hit rates. In PC trials, reaction times were lower at the cued location compared to the other locations (Cu: RT_hits_ = 926+/−59 ms; Uc_av_: RT_hits_ = 996+/−65 ms), although this difference was not significant (p = 0.380; ANOVA, Supplementary Fig. S1). Nevertheless, cueing produced clear attention effects on reaction times: RTs were significantly lower in PC, as compared to PE, trials at the cued location (Cu: AE(RT_hits_) = −122+/−35 ms, p < 0.001; Fig. 2F, Supplementary Fig. S1), and were significantly higher in PC, as compared to PE, trials at the uncued locations (Co_av_: AE(RT_hits_) = 107+/−33 ms, p < 0.001). Again, the cue-ipsilateral location was an exception to these trends. Cueing did not significantly modulate reaction times at the Ip location (Ip: AE(RT_hits_) = 36+/−41 ms, p = 0.197; Fig. 2F).

Finally, we measured the visual interference effects of exogenous cueing, as quantified by the difference in behavioral metrics across PE and PL trials. Cueing produced a weak, suppressive visual effect on hit rates (Cu: VE(hits) = −5.2+/−2.4%, p = 0.049; Fig. 2D), and an increase in reaction times at the cued location (VE(RT) = 87+/−49 ms p = 0.030; Fig. 2F), but not at the other locations (VE(hits) p = 0.981; VE(RT) p = 0.971). Nor was there an effect of visual interference on correct rejection rates (p = 0.666) or false alarm rates at any location (p = 0.615).

Taken together, these results indicate that exogenous cueing produced mixed effects on hit rates, false-alarm rates and reaction times. Specifically, cueing produced significantly higher hit rates and false-alarm rates at the cued location, compared to other locations in the pre-cued (PC) trials. Moreover, computing attention effects (AE) – by comparing pre-cue with early post-cue trials – revealed benefits in terms of both hit rates and reaction times, but only at the cued location. Conversely, computing attention effects at the uncued locations revealed a mixed pattern of costs across visual hemifields. We sought to synthesize these disparate, patterns of cueing effects using the framework of signal detection theory.

### Global and local effects of exogenous cueing on sensitivity

**Figure 3:**
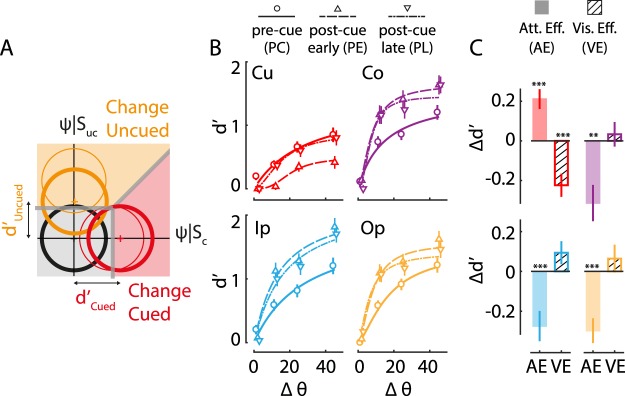
Localized attentional effects of exogenous cueing on sensitivity. (**A**) Schematic of m-ADC model depicting exogenous cueing effects on sensitivity (d′). Thin circular contours represent multivariate decision variable distributions whose means correspond to sensitivity values at baseline; thick contours represent changes in sensitivity due to exogenous attention at the cued and uncued locations. Other conventions are as in Fig. 1C. (**B**) Psychophysical functions (data pooled across n = 45 subjects) showing sensitivity, estimated from the m-ADC model, at different orientation change magnitudes (Δθ) for the four locations (Cu, Ip, Co, Op) and three trial types (PC, PE, PL). Curves: Naka-Rushton fits. Other conventions are as in Fig. 2B. (**C**) Modulation of sensitivity (averaged across orientation change values) by exogenous cueing for the four locations (Cu, Ip, Co, Op). Filled bars: Attention effect. Hatched bars: Visual effect. Other conventions are as in Fig. 2D.

We asked if these mixed effects of exogenous cueing on behavioral metrics could be resolved by dissecting the effects of cueing into sensitivity and bias components. To estimate sensitivity and bias from the 5 × 5 stimulus response contingency table, we employed a multidimensional signal detection model, the m-ADC model (Fig. 3A)^28,29^. The model is parameterized by sensitivity and criterion parameters, with one pair of parameters at each location (see Supplementary Methods). Sensitivity (d′) at each location represents the detectability of signal in noise (change versus no change) at each location. On the other hand, the detection threshold (c) at each location represents a threshold for sensory evidence for reporting a change at each location (for a full description, see Methods).

Before estimating cueing effects on sensitivity and bias, we tested the validity of the model for explaining subjects’ behavioral data in this multialternative task. We performed a randomization goodness-of-fit test, based on the chi-squared statistic (see Supplementary Methods). The goodness-of-fit tests yielded p-values that ranged between 0.60 and 0.99 (mean+/−stderr = 0.86+/−0.01) across subjects indicating that the model fits did not deviate significantly from the data (Supplementary Fig. S1).

We examined the effect of exogenous cueing, first, on sensitivity (this section) and, next, on choice bias (next section). Overall, sensitivity at the cued location was lower than the average sensitivity at the uncued locations, in all three conditions (Supplementary Fig. S2; p < 0.001, signrank test), although these differences were not significant when we compared sensitivity at the cued location with each of the uncued locations (p > 0.5, n-way ANOVA, Tukey-Kramer correction for multiple comparisons). We hypothesized that this overall trend of sensitivity decrease at the cued location occurred due to visual interference effects of the cue, and that isolating the attentional effect of the cue would yield an increase in sensitivity.

Indeed, isolating the attentional effect of exogenous cueing – by comparing sensitivity in the pre-cued (PC) trials, as compared to the early post-cue (PE) trials – revealed a gain in sensitivity at the cued location (Fig. 3B,C) (Cu: AE(d′) = 0.21+/−0.05, p < 0.001). However, in stark contrast to the mixed effects on hit rates (Fig. 2D), the attentional effect of exogenous cueing was to decrease sensitivity, uniformly, at all uncued locations (Ip, Co, Op): d′ was significantly lower in the PC trials (Fig. 3B, solid lines) as compared to PE trials at all uncued locations (Uc_av_: AE(d′) = −0.30+/−0.06, p < 0.001; Fig. 3B,C). The magnitude of the attentional effect of cueing was not significantly different across uncued locations (p = 0.886; Supplementary Fig. S2).

Next, we quantified the visual interference effect of exogenous cueing on sensitivity (VE(d′); Fig. 3C). We found a strong reduction in d′ indicating suppression of sensory information processing by the cue: average sensitivity in the PE trials was significantly lower than that in the PL trials at the cued location (Cu: VE(d′) = −0.22+/−0.06, p < 0.001). In contrast, at all other uncued locations, the visual interference effect of the cue was minimal or absent (Uc_av_: VE(d′) = 0.06+/−0.04, p = 0.108). This pattern of visual interference effects, furthermore, suggested that subjects were performing the task based on their perception of orientation changes rather than by encoding gratings into working memory: because the gratings were presented on the screen for an extended duration, encoding the gratings into working memory would have rendered visual interference by the cue ineffective (for details see Supplementary Results, section on “*Perceptual versus working memory strategies for localizing changes*”). We also performed additional control analyses to confirm that these visual interference effects were comparable between pre-cue and early post-cue conditions (see Supplementary Results section on “*Testing for visual interference effects in pre-cue versus early post-cue conditions*.”).

Finally, we asked whether these effects of exogenous cueing on d′ depended on cue contrast. Specifically, we tested the hypothesis that cues of lower contrast would produce a weaker visual suppression effect. One-third (15/45) of our participants were tested with full (100%) contrast exogenous cues whereas the remaining two-thirds (30/45) were tested with half (50%) contrast cues. Visual interference effects resulted in a strong and significant reduction in d′ at the cued location for full contrast cues (Cu: VE(d′) = −0.31+/−0.08, p = 0.002, Uc_av_: VE(d′) = 0.08+/−0.06, p = 0.252; Supplementary Fig. S2), but resulted in a comparatively weaker reduction in d′ for half contrast cues (Cu: VE(d′) = −0.18+/−0.08, p = 0.019, Uc_av_: VE(d′) = 0.06+/−0.05, p = 0.271; Supplementary Fig. S2). Interestingly, the two types of cues produced complementary patterns of attentional benefits and costs across cued and uncued locations. Full contrast cues produced a non-significant improvement in d′ at the cued location (Cu: AE(d′) = 0.10+/−0.06, p = 0.073; Supplementary Fig. S2) but a strong reduction in d′ at all uncued locations (Ip: AE(d′) = −0.46+/−0.14, p = 0.002, Co: AE(d′) = −0.60+/−0.12, p < 0.001, Op: AE(d′) = −0.67+/−0.09, p < 0.001). On the other hand, half-contrast cues produced a significant improvement in d′ at the cued location (Cu: AE(d′) = 0.26+/−0.07, p < 0.001; Supplementary Fig. S2), but only a weak reduction in d′ at uncued locations (Ip: AE(d′) = −0.18+/−0.09, p = 0.008, Co: AE(d′) = −0.2+/−0.1, p = 0.185, Op: AE(d′) = −0.11+/−0.06, p = 0.060). This global attentional effect, and the local visual suppression effect, of exogenous cueing, were supported by two other lines of evidence, which are outlined in the Supplementary Results (section on *Supporting evidence for attention and visual suppression effects of exogenous cueing)*.

Taken together, these results indicate that, although sensitivity at the exogenously cued location was lowest compared to other locations, the attentional effect of exogenous cueing − computed by comparing sensitivity in pre-cue versus post-cue trials (Fig. 1B) − was consistent with a global reallocation of sensory processing resources from uncued locations toward the cued location. Thus, exogenous cueing produced two distinct effects on d′: (i) a global attentional effect involving a benefit in d′ at the cued location and a corresponding cost at all uncued locations, and (ii) a local, visual interference effect involving suppression of d′ at the cued location alone. The visual interference effect was mitigated with cues of lower visual contrasts.

### Hemifield-specific effects of exogenous cueing on choice bias

**Figure 4:**
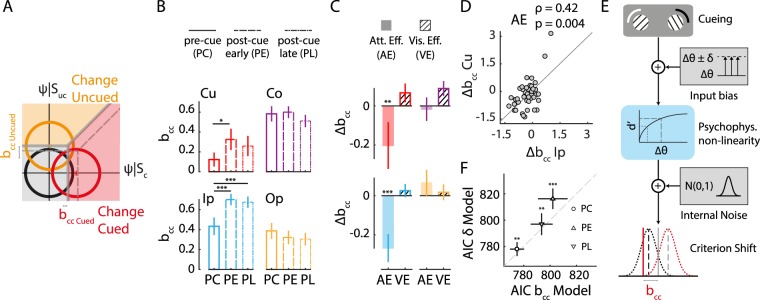
Hemifield-specific attentional effects of exogenous cueing on choice bias. (**A**) Schematic of m-ADC model depicting exogenous cueing effects on choice bias (b_cc_). Dashed gray lines: Decision boundaries at baseline. Solid gray line: Change in decision boundaries due to exogenous attention. Other conventions are as in Fig. 1C. (**B**) Choice bias for the four locations (Cu, Ip, Co, Op) and three trial types (PC, PE, PL). Other conventions are as in Fig. 2C. (**C**) Modulation of choice bias by exogenous cueing for the four locations (Cu, Ip, Co, Op). Filled bars: Attention effect. Hatched bars: Visual effect. Other conventions are as in Fig. 2D. (**D**) Covariation between attentional modulations of choice bias (AE(b_cc_)) at the cued (Cu) and cue-ipsilateral (Ip) locations. Circles: data representing individual subjects; line: best linear fit. (**E**) Schematic of the input gain model, in which exogenous cueing effect on bias arises from a gain in sensory input (Δθ ± δ; upper right inset) and choice bias model, in which the effect arises from a shift in the choice criterion (b_cc_; lowermost panel) (see text for details). (**F**) Akaike Information Criterion (AIC) for the input gain model (y-axis) versus the choice bias model (x-axis). Circle, upright and inverted triangles: AIC values for PC, PE and PL trials. Error-bars: s.e.m. across subjects. Dashed gray line: line of equality.

Next, we investigated the effect of exogenous cueing on choice bias (Fig. 4A) We quantified choice bias with the choice criterion (b_cc_), computed using the detection threshold and sensitivity parameters estimated with the m-ADC model (Methods). The value of the choice criterion has an inverse relationship with choice bias: lower the choice criterion value at a location, higher is the bias for reporting a change at that location.

First, we quantified the effect of exogenous attention on bias (AE(b_cc_)). Exogenous cueing significantly increased bias at the cued (Cu) location (Fig. 4B,C), in that, b_cc_ was significantly lower in the PC trials as compared to the PE trials (Cu: AE(b_cc_) = −0.20+/−0.12, p = 0.005). On the other hand, exogenous attention produced an interesting pattern of bias changes at the uncued locations. Bias increased at the Ip location in the PC trials (Fig. 4B, solid lines) as compared to the PE trials (Fig. 4B, dashed lines), indicating a strong attention effect of exogenous cueing at this location (Ip: AE(b_cc_) = −0.27+/−0.07, p < 0.001, Fig. 4C). In contrast, no significant change in bias occurred at the other two uncued locations (Co_av_: AE(b_cc_) = 0.02+/−0.05, p = 0.722). The magnitude of bias modulation was equivalent across Cu and Ip locations (p = 0.511) and was significantly greater than that at both Co or Op locations (p < 0.001). Furthermore, we found that bias modulations at the Cu and Ip locations were correlated (Fig. 4D, r = 0.42; p = 0.004). These results indicate that exogenous pre-cueing produced an increase in bias, relative to post-cueing, only for locations in the hemifield ipsilateral to the cue. Moreover, the correlation in bias modulations across these locations (Cu, Ip) suggests that a common mechanism mediated the enhancement of bias across the cued-hemifield.

Second, we quantified the visual interference effect of exogenous cueing on bias VE(b_cc_), (Fig. 4C). We found no measurable effect of visual interference on bias at any location: Bias changes in PE and PL trials were not different from each other (Cu: VE(b_cc_) = 0.07+/−0.05, p = 0.177; Uc_av_: VE(b_cc_) = 0.04+/−0.03, p = 0.126). These results indicate that exogenous post-cueing effects on bias were not different, regardless of the latency of cue appearance, following the change.

Third, we compared the bias across locations in the pre-cueing and post-cueing conditions. We found that bias was uniformly highest at the cued location during the PC trials (p < 0.001; Supplementary Fig. S3). In particular, the value of b_cc_ was not significantly different from zero at the cued location in pre-cueing (PC) trials (Fig. 4B). A b_cc_ value of zero corresponds to the optimum criterion for detection under uniform priors (of signal and noise) and uniform rewards for each correct response^32^. The results thus indicate that subjects placed their criterion close to the theoretical optimum at the cued location on PC trials. In contrast, in the post-cueing trials (PE, PL) b_cc_ values were significantly greater than zero at all locations, indicating sub-optimal decisions. Moreover, in these trials (PE, PL) bias at the cued location was significantly higher than that at the ipsilateral and contralateral locations (p < 0.001), and these differences in bias – between the cued location and ipsilateral and contralateral locations – were strongly correlated (PE: r = 0.933, p < 0.001; PL: r = 0.868, p < 0.001; Supplementary Fig. S3).

Finally, we sought to distinguish whether these choice bias modulations reflected shifts in decision criteria (choice bias), changes of sensory input gain or had a motoric origin (response bias). The control analyses, described in the Supplementary Results (section on *Distinguishing between choice bias*, *sensory input gain and motoric response bias)*, revealed strong model evidence in favor of shifts of decisional criteria over alternative interpretations (Fig. 4E,F).

To summarize, exogenous cueing produced the highest choice bias at the cued location compared to the other locations. The effect of exogenous attention − as quantified by the difference in bias between pre-cue and post-cue trials − revealed an increase in choice bias for stimuli at locations ipsilateral (Cu, Ip), but not contralateral (Co, Op), to the exogenous cue. The increases in bias at the cued and ipsilateral locations were correlated, indicating that these bias effects were mediated by a common neural mechanism (see Discussion). Neither sensory input bias nor motoric response bias could explain these attentional effects indicating that these reflected modulations of choice bias (shifts of choice criteria) by exogenous attention.

### A synthetic model of exogenous attention’s effects on sensitivity, bias and reaction times

**Figure 5:**
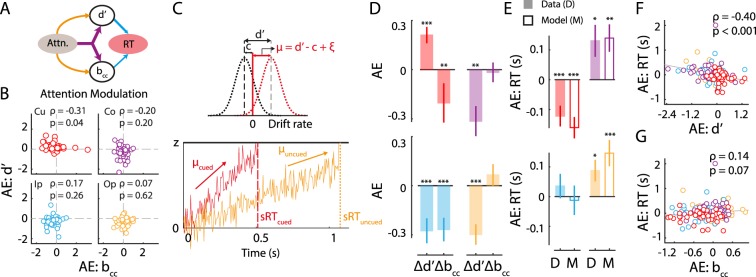
Diffusion decision model linking exogenous cueing effects on sensitivity, bias with reaction times. (**A**) Schematic depicting common (purple arrows) and dissociable (orange arrows) mechanisms by which exogenous attention could modulate sensitivity (d′) and choice bias (b_cc_). Bias and sensitivity, in turn, impact reaction times. (**B**) Covariation between attentional modulations of sensitivity (AE(d′)) and bias (AE(b_cc_)) at each of the four locations. Data points: Individual subjects. ρ and p values for each correlation are indicated in the upper right corner of each sub-panel. Other conventions are as in Fig. 2B. (**C**) Schematic of Diffusion Decision Model (DDM) that relates reaction times to sensitivity and bias. (Top) Schematic of relationship between drift rate (μ) in DDM and sensitivity (d′) and criterion (c) in the m-ADC model; μ = (d′ - c + ξ) where ξ is an offset parameter (see Methods). (Bottom) Schematic simulated runs of evidence accumulation when a change occurred at the cued location (red) or at the uncued location (orange). In each case, the reaction time (sRT; dashed or dotted vertical line) corresponds to the time at which the accumulated evidence crossed a threshold or bound (z). (**D**) Attention effect of exogenous cueing (AE) (filled bars) on sensitivity (d′) and bias (b_cc_) (replotted, for convenience of comparison). Other conventions are as in Fig. 2D. (**E**) Attention modulation of reaction times (AE(RT)) computed from model simulations (M; unfilled bars) showed close agreement with that computed from the observed data (**D**; filled bars), at all locations. Error-bars: s.e.m. Other conventions are as in Fig. 2D. (**F**) Covariation of attentional modulation of RT (AE(RT)) with attentional modulation of sensitivity (AE(d′)). Circles: Data for each subject; colors represent values at each location. Line: best linear fit. Other conventions are as in panel (B). (**G**) Same as in panel F, but for covariation of attentional modulation of reaction times (AE(RT)) with attentional modulation of bias (AE(b_cc_)). Other conventions are as in panel (B).

The distinct patterns of sensitivity and bias effects produced by exogenous cueing suggest distinct mechanisms underlying the modulation of these sensory and decisional components. To further evaluate this hypothesis we tested, first, whether sensitivity and bias changes were correlated at each location. Sensitivity changes (AE(d′)) were not significantly correlated with bias changes (AE(b_cc_)) at any of the four locations across subjects (Fig. 5B; following Benjamini-Hochberg correction for multiple comparisons). Next, we tested whether modulations of sensitivity and bias were correlated across task blocks. For this, we performed a split half analysis of the data, by testing whether a change in AE(d′) was correlated with the change in AE(b_cc_), across the two halves of the data (first three versus last three blocks). No significant correlations were observed in the modulations of sensitivity and bias across blocks (Supplementary Fig. S4). We also confirmed that these null results were not due to a lack of reliability in each metric computed separately (Supplementary Results, section on “*Testing for reliability of sensitivity and bias modulations”*). These results suggest that distinct mechanisms mediate sensitivity and bias modulations produced by exogenous cueing (Fig. 5A).

Next, we asked how these distinct sensitivity and bias modulations, each, influenced the dynamics of perceptual decisions in this task. In particular, we sought to explain, within a synthetic framework, the disparate patterns of changes in sensitivity, bias and reaction times (RT) produced by exogenous cueing. For this, we availed of the diffusion decision model (DDM), which combines signal detection theory with the drift diffusion model, tying together concepts of sensitivity, bias and reaction times in terms of a diffusion-rate and a decision threshold^36^. According to DDM, decision-making is modeled as a stochastic evidence accumulation (diffusion) process. The stopping time of the evidence accumulation process corresponds to the reaction time. The drift-rate of the process captures the rate of evidence accumulation and is related to the d′ value: higher the d′ value, faster the evidence accumulation, and faster the reaction time. In our model, the criterion defines the origin of the drift rate, such that lower the criterion, higher the net drift rate at that location (Fig. 5C)^36,41^. Thus, lower the decision criterion, higher the bias, and faster the reaction time. When the accumulated evidence reaches the decision threshold (Z), a decision in favor of reporting the change is made. Although the DDM has not been formally extended to multialternative m-ADC tasks^41^ we sought to explain qualitative trends in the data using multiple one-dimensional DDM models (race model).

As a preliminary step, we plotted the attention effect of exogenous cueing (AE) on sensitivity, bias and reaction times alongside each other (Fig. 5D, replotted from Figs 3C, 4C and 5E, left bars, replotted from Fig. 2F, respectively). The data indicated that exogenous attention’s effects on in reaction time, d′ and b_cc_ were disparate, but could be explained within the DDM framework. Because the attention effect on d′ and bias was highest at the cued location, corresponding to a higher drift rate at this location, RTs were fastest at this location (Fig. 5E top-left). In contrast, because cueing produced a reduction in d′ (slower drift) at the Co and Op location but did not affect bias, RTs were slower at these locations (Fig. 5E, right). Finally, at the Ip location, cueing produced a reduction in d′ but a gain in bias, which potentially canceled out each other’s effects, thereby resulting in no net effect on RT (Fig. 5E bottom-left). We tested these hypotheses by performing simulations using the DDM model (Methods). Simulated effects of exogenous cueing, based on the DDM, showed striking qualitative similarities to those observed in the data, confirming a putative mechanistic link between sensitivity, bias and RT effects of exogenous cueing (Fig. 5E, right bars).

Finally, we tested which of the parameters, d′ or bias, produced a greater effect on RT in the data. We observed that AE(RT) was significantly correlated with AE(d′) (ρ = −0.40, p < 0.001) but not AE(b_cc_) (ρ = 0.12, p = 0.12; Fig. 5F,G). These trends were further confirmed with several additional control analyses (Supplementary Results, section on “*Effect of sensitivity and bias on reaction time effects of exogenous cueing”*).

To summarize, simulations showed that exogenous attention’s effects on reaction time could be qualitatively explained through the DDM model, linking reaction times with sensitivity and bias. Quantitatively, exogenous attention’s effects on sensitivity, rather than bias, were strongly predictive of benefits on reaction times (Fig. 5A). Taken together these results provide a synthetic model of sensitivity and bias effects on reaction times, and support a dissociation between sensitivity and bias effects of exogenous cueing.

## Discussion

When a salient stimulus automatically captures our attention, does it enhance perception (sensitivity) at the attended location, does it prioritize choices (bias) at that location, or both. To answer this question, we designed a multialternative change detection task with exogenous cueing, and analyzed our data with a multidimensional signal detection model.

Overall, exogenous cueing produced a strong, suppressive effect on sensitivity that was localized to the cued location (Fig. 3B,C, VE). This suppressive effect occurred, likely, because of the presence of a high-contrast cue proximal to the stimulus at the cued location, which interfered with sensory processing associated with detecting a change in stimulus orientation. Having accounted for this visual interference effect, our analysis revealed that the attentional effect of exogenous cueing – obtained by subtracting pre-cue from post-cue effects –was to produce a systematic gain in sensitivity, specifically at the cued location (Fig. 3C, AE). In contrast, even without controlling for visual interference effects, bias was unequivocally the highest (lowest b_cc_) at the cued location relative to other locations on pre-cued trials (Fig. 4B, solid red line), indicating that cueing produced a robust gain in bias. Moreover, this gain in bias occurred, in a correlated manner, at both the cued and cue-ipsilateral locations (Fig. 4C,D, AE). Furthermore, sensitivity and bias modulations produced by exogenous cueing were uncorrelated (Fig. 5B), and produced dissociable effects on reaction times (Fig. 5F). To our knowledge, our study is the first to address how exogenous attention modulates sensitivity and bias to produce systematic effects on behavioral accuracies and reaction times.

The results suggest that exogenous cueing produces not a unitary “attention field”, but a localized “sensitivity field” and a more diffuse “bias field”^42^. In the real-world, the diffuse choice bias could reflect the need for assigning decisional priority to information not only at the location of the salient stimulus that captured attention, but also for other stimuli in its vicinity (same visual hemifield). This hypothesis, and its neural substrates, must be investigated in future studies.

These effects of exogenous cueing were revealed by analyzing behavior with a multidimensional signal detection model: the m-ADC model^28^. Our choice of using a multialternative change detection task and m-ADC model was motivated by several key reasons. First, this task permitted measuring behavioral effects of exogenous cueing at multiple locations in the visual field, at various positions relative to the cue. Yet, measuring cueing effects in terms of “raw” behavioral metrics such as accuracies (hit rates), false-alarm rates and reaction times produced mixed, puzzling trends, which were not readily explained (Fig. 2D). By contrast, analysis with the m-ADC model permitted quantifying these trends in terms of sensitivity and bias effects, for which the trends were neatly resolved (Figs 3C and 4C). Second, our choice of a change detection (and localization) task, rather than a response probe task (e.g. Wyart *et al*.^43^) was motivated by recent literature, which suggests that bias in response probe tasks is linked to signal expectation (priors) rather than attention mechanisms^39^. Our detection and localization task, and m-ADC model, permitted quantifying a spatial detection bias at each location, and its modulation by exogenous attention. Third, we could have also used a simple detection task, for example, involving the detection of a low contrast target presented at one of four locations marked by pedestals^4^; the provision of such pedestals reduces uncertainty associated with stimulus location. Nevertheless, design choices regarding the shape and contrast of the pedestals may critically affect the exogenous cue’s ability to modulate the detection of the low contrast target. In our change detection task, all gratings were presented at full contrast, thereby minimizing stimulus location uncertainty, while the psychometric function was measured by varying the orientation change angle (rather than contrast). Finally, several recent studies^28–30,44,45^ have used a change detection paradigm similar to ours to measure the effect of endogenous cueing of attention. Employing a similar change detection task design in our study permitted us to readily compare the effects of endogenous and exogenous cueing on sensitivity and bias.

There is an active debate in literature over whether exogenous and endogenous cueing of attention share the same or different neural mechanisms^46,47^. Studies comparing endogenous and exogenous attention’s effects on neurons and behavior have reported mixed results^4,16,48–52^. Similarly, functional imaging studies have provided evidence for common^47^, overlapping^19^ or distinct^53^ neural substrates of the two types of attention. Our recent work^29^ investigated the effects of endogenous cueing of attention, on sensitivity and bias, with a multialternative change detection task and a stimulus configuration similar to the one used in the present study. Comparing exogenous and endogenous cueing effects on multialternative decision-making revealed many similarities but also certain key differences with regard to how the two types of attention operate.

At first glance, several important similarities were apparent. First, endogenous attention produced a gain in sensitivity, which was spatially localized to the cued location; exogenous cueing’s attentional effect (comparing pre-cue and post-cue trials) also yielded a gain in sensitivity at the cued location (Fig. 3C). In both cases, sensitivity was modulated in a response gain-like manner, consistent with a smaller attention field compared to stimulus size^51^. Second, the bias produced by pre-cueing was the highest at the cued location for both types of attention. Third, both exogenous and endogenous attention produced decisions that were more optimal at the cued location. In the case of endogenous cueing indices of optimal decision-making were systematically higher at the cued location^29^, while in the case of exogenous cueing optimal values of choice criteria occurred, specifically, at the cued location. Finally, both endogenous and exogenous attention produced uncorrelated modulations of sensitivity and bias, indicating that sensitivity and bias effects are dissociable and likely to be mediated by distinct mechanisms for the two types of attentional cueing.

Nevertheless, several key differences were also observed. First, exogenous cueing of attention produced a strong cost (reduction) for sensitivity at the uncued locations relative to baseline; the same was not observed with endogenous cueing^29^. Second, while exogenous cueing modulated attentional bias in a hemifield specific manner, primarily producing an increase in bias at the locations ipsilateral to the cued hemifield, endogenous cueing modulated bias in a graded manner that depended on cue validity. Third, reaction time effects of exogenous cueing were strongly correlated with sensitivity, rather than with bias, whereas the reverse was true for reaction time effects of endogenous cueing^29^.

In summary, while exogenous and endogenous cueing may engage some shared mechanisms, there are also likely to be significant differences. We propose that rather than investigating common or distinct neural substrates of exogenous and endogenous attention, it may be more relevant to investigate whether neural substrates of each component of attention (sensitivity and bias) are shared or distinct.

## Methods

### Participants

50 subjects (22 female, age range 18–43 yrs, median age 23 yrs) with no known history of neurological disorders, and with normal or corrected-normal vision participated in the main experiment. All participants provided written informed consent, and experimental procedures were conducted according to the protocols approved by the Institute Human Ethics Committee at the Indian Institute of Science, Bangalore. Five subjects were excluded from analysis because of improper gaze fixation (see Supplementary Methods section on *Eye Tracking*). Data from 45 subjects were included in the final analysis.

### Experimental design

Participants were tested on a cued, five-alternative change detection task. Subjects were seated in an isolated room, with the head positioned on a chin-rest 60 cm from the center of a contrast calibrated visual display (22-inch DELL LCD monitor; contrast calibrated with an X-Rite i1 Spectrophotometer). Stimuli were programmed with Psychtoolbox (version 3.0.11) using MATLAB R2016b (Natick, MA). Responses were recorded with an RB-840 response box (CedrusInc). Fixation was monitored with a 60 Hz infrared eye-tracker (Gazepoint GP3).

We employed a previously published 4-ADC task design^29,30^ with exogenous cueing. Subjects began the task by fixating on a fixation cross (0.5° diameter) at the center of a grey screen. 1500 ms after fixation cross onset, four full-contrast, sine-wave gratings appeared, one in each visual quadrant at a distance of 5° from the fixation cross (grating diameter: 2° visual angle, spatial frequency, 3.5 cycles/degree). The orientation of each grating was drawn from a uniform random distribution, independent of the other gratings, and pseudorandomized across trials. Concurrently with the grating stimuli 4 cue placeholders (dark, full negative contrast, arcs) were presented, each adjoining one stimulus. The center of each placeholder was at ±45° relative to the horizontal, and 6.7° visual angle from the fixation cross center. Each arc subtended 1.3° visual angle, with a thickness of 0.4° visual angle. The center of each placeholder was separated from the edge of the adjacent grating by a distance of 0.9° visual angle (Fig. 1A).

After a variable delay following stimulus presentation (600 ms-2200 ms, drawn from an exponential distribution), in one-third of the trials (pre-cue or PC trials), the contrast of one of the 4 placeholders (randomly chosen) was transiently increased (for 50 ms) to a high value (50% or 100% positive contrast, see below), and reverted to baseline, mimicking a bright flash; this flash corresponded to the exogenous cue. After 17 ms, the gratings were blanked for 50 ms and then reappeared. Upon reappearance, either any one of the four gratings had changed in orientation, or none had changed. Subjects indicated the location of change (one of four locations), or not having detected the change at any location, by pressing one of five buttons on a response box (Supplementary Fig. S1). In the remaining two-thirds of the trials (post-cue), the cue appeared at one of two delays, either at 17 ms (“early post-cue”, PE) or 117 ms (“late post-cue”, PL) after grating reappearance. The rationale for the cue timings for these control conditions (post-cue trials) are described in a later section on *Computing attention and visual interference effects* (Fig. 1B). As in the pre-cue trials, subjects provided their response, by pressing one of five buttons.

We term trials in which a change in orientation occurred at one of the four locations as “change” trials (80% of all trials), and trials in which no change in orientation occurred as “no change” trials (20% of all trials). We term the location closest to which the cue occurred as the “Cued” (Cu) location, the uncued location diagonally opposite to the cued location, as the “Opposite” (Op) location, and the two other uncued locations as “Ipsilateral” (Ip) or “Contralateral” (Co) locations, depending on whether they were in the visual hemifield ipsilateral or contralateral, respectively, to the cued location (Fig. 2A). Changes were equally and pseudo-randomly distributed among these four locations. Thus, the cue was uninformative spatially and had a conditional validity of 25% on change trials among the four locations. The experiment was run in six blocks of 60 trials each (total, 360 trials per subject), with no feedback. In a training session prior to the experiment, subjects were familiarized with the task and response protocol for 1–2 blocks (60 trials each), with explicit feedback provided at the end of each trial about the location of the change and the correctness of their response. Data from these “training” blocks were not used for further analyses.

We tested three versions of the above task protocol, with slight variations. In the first protocol, the exogenous cue was presented at full (100%) contrast with no time limit given to the subjects for response (n = 15 subjects). The second variant was identical with the first protocol, except that the exogenous cue was presented at 50% contrast (n = 16). The third variant was identical with the second protocol, except that subjects were required to respond within a maximum window of 1250 ms (n = 14). Results were closely similar for all protocols and, unless otherwise indicated, data were pooled across protocols.

### m-ADC psychophysical model

Previous studies have employed one-dimensional signal detection models to distinguish sensitivity from bias in detection and localization tasks^26,54^. However, such an approach represents a model misspecification for analysis of behavior in multialternative localization tasks, as elaborated in Sridharan *et al*., 2014^28^. We applied a recently developed multidimensional signal detection model, the m-ADC model to analyze subjects’ behavior in this 5-alternative task. The m-ADC model overcomes key pitfalls of previous approaches by modeling the decision on each trial in a multidimensional decision space. Specifically, for the 4-ADC model used in this study, sensory evidence for the change event is represented along four orthogonal decision variable axes, one corresponding to each location. Decision surfaces are represented as manifolds comprising of 10 intersecting hyperplanes in a 4-dimensional space^28^. The hyperplanes belong to the family of optimal decision surfaces for distinguishing signals of each class (a change event at each location) from noise (no change), and for distinguishing signals of each class from the other (a change event at one location from a change event at another location). These hyperplanes are parametrized by four criteria (c), which reflect detection thresholds for reporting change versus no change at each location. In addition, the model is parameterized by four sensitivity (d′) values for each signal strength tested at each location, and are quantified as the distance between the means of the respective signal and noise distributions, measured in units of noise standard deviation, along the corresponding decision variable axis (Fig. 1C).

The model decouples and separately quantifies sensitivity and choice criteria at each location based on a 5 × 5 stimulus-response contingency table (Supplementary Methods) obtained from the multialternative task (Fig. 2A, Supplementary Fig. S1). A description and mathematical derivation of the model are provided in Sridharan *et al*., 2014; 2017^28,30^. An extension of the m-ADC model to tasks that seek to measure the entire psychophysical function at the cued and uncued locations is provided in Banerjee *et al*.^29^, and this latter model is used for behavioral analysis in this study (see Supplementary Methods section on *Model parameter estimation and goodness-of-fit*).

### Psychometric and psychophysical functions

To compute psychometric function (percent correct as a function of orientation change angle), we calculated the proportion of trials in which the subject detected and localized the change accurately; this was computed separately for each location (relative to the cue) and each trial type (PC, PE, PL). Percent correct values, across all angles of orientation change, were fitted with a three parameter sigmoid function to generate the psychometric function (Fig. 2B). Psychometric functions for all subjects were estimated with a set of four change angles spanning 2° to 45° ([2,12,25,45°]), presented in an interleaved, pseudo-randomized order across locations. The middle two angles (12° and 25°) were tested twice as often as the extreme two angles (2° and 45°). The population psychometric curve was generated by pooling responses across all subjects and computing the above metrics for the pooled data. False alarm and correct rejection rates were calculated, respectively, based on subjects’ incorrect and correct responses during no-change trials. Unless otherwise indicated, error bars represent standard error of the mean.

For each trial type (PC, PE, PL), subject’s responses were aggregated into a 5 × 5 stimulus-response contingency table, with each row and column representing, respectively, change events and responses at locations relative to the cued location (Fig. 2A). These are, in order: (i) the cued location (Cu), (ii) the location in an adjacent quadrant, ipsilateral (Ip) or (iii) contralateral (Co) to the cue, and (iv) the location in the quadrant diagonally opposite to the cue (Op). No change events and responses constitute the fifth row and column of the table. The behavioral responses in the 5 × 5 contingency table were analyzed with a multidimensional signal detection model, the m-ADC model^28–30^, to quantify psychophysical parameters (sensitivity and criteria) at each location, and for each trial type (detailed in Supplementary Methods, section on “*Model parameter estimation and goodness-of-fit”*). We then computed the mean sensitivity across change angles (d′_av_), as well the choice criterion (b_cc_ = c − d′/2), a measure of bias^28–30^.

The psychophysical function of sensitivity was generated by fitting sensitivity values across angles at each location with a three-parameter Naka-Rushton function. Asymptotic sensitivity (d_max_) and orientation change value at half-max (Δθ_50_) were fit as free parameters, whereas the slope parameter (n) was fixed at 2.0 based on pilot fits to the data. As before, a single combined psychophysical curve was generated by pooling contingencies across all subjects and fitting the m-ADC model to the pooled data (Fig. 3B).

We sought to quantify the effect of exogenous cueing on psychometric (e.g. hit rates, false-alarm rates) and psychophysical (e.g. d′, b_cc_) parameters, and to dissociate the effects of attention and visual interference due to the cue. Therefore, we analyzed our data in two steps. First, we investigated how each psychophysical measure (d′ or b_cc_) varied in each condition across the four locations (e.g. Fig. 3B). Next, we computed the attentional modulation effects and visual interference at each location as described next.

### Computing attention and visual interference effects

We matched the PC, PE and PL trial types in terms of the net visual input (but not the timing) associated with the exogenous cue. We specified different times of the cues for each trial type as follows: We treat the mid-point of the blank epoch to be “zero- time” (t0) (Fig. 1B, dotted vertical line) for the change event. This is meaningful because the blank is the key epoch that separates information relevant to detecting the change. In other words, the blank epoch separates the first set of gratings (before the blank) from the second set (after the blank). On pre-cue (PC) trials, the onset of the cue occurred 92 ms before t0, and on post-cue (early/PE) trials, the offset of the cue occurred 92 ms after t0. Similarly, On pre-cue (PC) trials, the offset of the cue occurred 42 ms before t0, and on post-cue (early/PE) trials, the onset of the cue occurs 42 ms after t0. Consequently, the mid-point of the cue presentation occurred either 67 ms before t0 (on PC trials) or 67 ms after t0 (on PE trials). The cue was, therefore, distributed symmetrically about t0, the zero time for the change event. In other words, pre-cue (PC) and early post-cue (PE) trials differed only in the order of presentation of the cue relative to the change event. When the cue preceded the change (PC trials) the cue produced both an exogenous attentional effect and a visual suppression effect^38^. On the other hand, when the cue followed, the change, it produced only a visual suppression effect. The cue was symmetrically distributed about the change event (t0) so that the visual suppression effect would be matched across the two types of trials (PC, PE). Therefore, subtracting psychophysical metrics in PE trials, from their values in PC trials, would uncover the residual attention effect of the exogenous cue (AE(m) = m_PC_ − m_PE_). Similarly, in the late post-cue (PL) trials, the cue occurred at a significantly later time point relative to the change (t0). In this case, the mid-point of cue presentation occurred 167 ms after t0, long enough for orientation-related sensory processing to be complete^55,56^. Therefore, visual interference effects should be significantly lower for these trials. Hence, subtracting psychophysical metrics in PL trials, from their values in PE trials, would uncover the visual interference effects of the exogenous cue (VE(m) = m_PE_ − m_PL_).

### Analysis of reaction times and simulations with the Diffusion Decision Model

Reaction times were quantified as the time from the onset of the change until the subject’s button press response. Reaction times were compared across cued and uncued locations with ANOVA and Tukey-Cramer post-hoc tests.

We simulated the effect of sensitivity (d′) and bias (b_cc_) on reaction-times (RT) using the Diffusion Decision Model (DDM; (Ratcliff *et al*.^41^) which models evidence accumulation as a noisy diffusion process. In the conventional drift-diffusion model, decision time is parameterized by an accumulation rate (or drift rate, μ), starting point (origin) and stopping threshold of the evidence accumulation process (z). DDM relates parameters in the drift-diffusion model, with the sensitivity and criterion parameters of signal detection theory. Here, we used a version of the DDM in which the drift rate was modeled as a linear combination of sensitivity and criteria (Ratcliff *et al*.^41^). Specifically, the sensitivity was directly related to the drift rate, and the criterion defined the origin of the drift rate at each location (Fig. 5C), such that a lower criterion corresponded to a higher, overall drift rate (μ = d′-c); this latter class of models has recently been evidenced in data from both humans and monkeys^57^. We also tested a model in which the criterion defined a stopping threshold, as is conventional in drift-diffusion models^41^, but found poorer qualitative fits to our data, across a range of model parameters. Our model contained 2 additional free parameters for fitting the data: an offset term for the evidence accumulation rate (ξ = 2.0) and a coefficient (k = 10^−3^) for the noise term, whose values were chosen to provide close fits to the data, although the results were robust to the choice of these parameters. The overall model can be summarized as follows:

$$\begin{array}{ccc}{{\rm{y}}}_{{\rm{i}}}({\rm{t}}) & = & {\rm{\Sigma }}({\mu }_{{\rm{i}}}{\rm{\Delta }}{\rm{t}}+{\rm{k}}\,{{\rm{w}}}_{{\rm{i}}{\rm{n}}}(t));\,{{\rm{w}}}_{{\rm{i}}{\rm{n}}}\sim {\rm{N}}(0,1)\\ {\rm{w}}{\rm{h}}{\rm{e}}{\rm{r}}{\rm{e}}\,{\mu }_{{\rm{i}}} & = & ({{\rm{d}}}_{{\rm{i}}}^{{\rm{^{\prime} }}}-{{\rm{c}}}_{{\rm{i}}}+\xi ),\\ {\rm{R}}{\rm{T}} & = & min\,\{t:\,{{\rm{y}}}_{{\rm{i}}}({\rm{t}}) > z\}\end{array}$$

where y_i_(t) denotes the accumulated evidence at the i^th^ location up to time t (i = 1,2,3,4), μ_i_ denotes the evidence accumulation rate and w_in_ denotes the noise added at each time step, drawn from a standard normal distribution (N(0,1)). The origin of evidence accumulation was set to zero and the decision threshold (z) was set to 1 at all locations. Thus, the model is akin to a “race” model with four competing evidence accumulators, one at each location, and the decision is made when one of the four accumulators crosses the decision threshold. The time step for the simulation was taken to be 1 millisecond, and accumulated evidence was calculated with the Euler method. A total of 288 trials (24 trials per location and condition) were used for the simulation, which matched our actual experimental task (only change trials were simulated). The average reaction time across trials was computed for each subject, location (Cu, Ip, Op, Co), and each trial type (PC, PE, PL), separately, based on the d′ and c for that subject, location and trial type. The attention effects and visual interference effects for the simulated RTs were then computed, with an identical procedure as for the experimental RTs.

### Statistical analyses

Tests of significant differences in sensitivity (Fig. 3B) across trial types were performed using an ANOVA treating locations and trial types as discrete factors and change angle as a continuous factor, with Tukey-Kramer correction for multiple comparisons. A similar ANOVA was performed for choice bias (Fig. 4B) without angles as a factor. Significance tests comparing attention or visual interference effects on different parameters for each location were performed using a Wilcoxon signed rank test with a Benjamini-Hochberg correction for multiple comparisons. Correlations across different parameter values were computed using robust correlations (“percentage-bend” correlations) to prevent outlier data points dominating the correlations^58^; correlation coefficient (ρ) and significance (p) values are indicated on the corresponding figures. Unless otherwise stated, significant p-values at the successively higher levels of significance (p < 0.05, p < 0.01 and p < 0.001) are shown with successively higher number of asterisks (* (single-star), ** (two-stars) and *** (three stars)), above the respective bar plots.

Details regarding eye-tracking, constructing contingency tables, model parameter estimation and goodness-of-fit, model comparison analysis and reaction time regression and prediction analysis are provided in the Supplementary Methods.

## Supplementary information

Supplementary Information

## Data Availability

Data and code for the analyses presented in the paper are available online at: 10.5061/dryad.s1725t6.
