# Supplementary material for: Dissociable sensitivity and bias mechanisms mediate behavioral effects of exogenous attention
Source: Sci Rep. 2019 Sep 2;9:12657. doi: 10.1038/s41598-019-42759-w (PMC6718663; doi:10.1038/s41598-019-42759-w)

Supplementary Information for:

Dissociable sensitivity and bias mechanisms mediate behavioral effects of exogenous attention

Authors:

Vishak Sagar, Ranit Sengupta, Devarajan Sridharan[†]

Supplementary Methods

Eye-tracking. Subjects' gaze was binocularly tracked (GP3 eyetracker, Gazepoint Inc., 60 Hz) and the deviation in their gaze from the fixation cross was recorded and stored for offline analysis. Trials in which the eye-position deviated by more than 2 degrees radially from the fixation cross from the onset of the cue (pre-cue trials) or onset of the blank (post-cue trials) until the final response were removed from further analysis. Our subjects were of South-Asian origin and exhibited dark pigmentation of the iris, rendering it challenging to distinguish from the pupil. The contrast of the pupil (relative to the iris) was weak, and the tracker occasionally lost the location of the pupil; trials in which this occurred for more than 100 ms continuously were also excluded from the analysis. We also excluded data from subjects for whom the combined rejection rate (eye deviation and lost tracking) exceeded more than 25% of all trials (5/50 subjects). The median rejection rate for subjects included in the analysis was 8.8% [0.0-22%] (median, 95% confidence intervals). We also tested with a randomization test, based on the chi-squared statistic (see below), whether these rejected trials significantly altered the distribution of responses in the contingency table for any subject, and found that this was not the case (p-values for distributions before and after rejection: 0.99, mean across subjects).

Contingency tables. Subjects' responses in the task were used to construct 5x5 stimulus response contingency tables, one for each of the pre- and post-cue trial types (PC, PE, PL). Change locations were represented on the rows and response locations on the columns; no-change events and responses

[†]Centre for Neuroscience, Indian Institute of Science, C. V. Raman Avenue, Bangalore, 560012, India. Correspondence and requests for materials should be addressed to D.S. (email: sridhar@iisc.ac.in)

were represented in the last row and last column respectively (Fig.2A). The contingency table was then restructured so that all change events and responses were measured relative to the cued location. Each contingency table comprised five categories of responses: hits, misses, false-alarms, mislocalizations and correct rejections (Supplementary Fig.S1). Since four values of orientation change were tested at each location, each contingency table contained 68 independent observations: 16 hits, 48 misidentifications and 4 false-alarms; the last category of responses do not depend (by definition) on orientation change magnitude.

Model parameter estimation and goodness-of-fit. To compute model parameters (sensitivity and bias), individual subjects' response contingencies were fitted with the m-ADC model [ref.28, main text]. Sensitivity is expected to change depending on the change angle magnitude ($\Delta\theta$); hence, different sensitivity values were estimated for each change angle tested. On the other hand, the criterion at each location was estimated as a single, uniform value across change angles, as the subject was not aware, apriori, of the change angle presented on each trial. Thus, the model estimated 20 parameters (d' values for each of the 4 locations and 4 angles, and criteria for each of the 4 locations) from 68 independent observations in the contingency table for each trial type. Sensitivities and criteria were estimated with maximum likelihood estimation (MLE), using a procedure described previously [ref.28, main text].

Goodness-of-fit of the model to the data was assessed using a randomization test based on the chi-squared statistic; the procedure is described in detail elsewhere [ref.29, main text]. A small p-value ($p<0.05$) for the goodness-of-fit statistic indicates that the observations deviated significantly from the model fit. The median p-values for PC trials was 0.90 (range: 0.68-1.00), for PE trials was 0.90 (range: 0.60-0.99) and PL trials was 0.83 (range: 0.61-0.98)(Supplementary Fig.S1), indicating that the model fit the observations well.

Split half analysis. To test whether the sensitivity modulation by exogenous attention at the cued and

uncued locations reflected the redistribution of limited sensory processing resources, we performed a split block analysis. Subjects performed 6 blocks of 60 trials each through the experiment. We split the experiment into the first three blocks and last three blocks and used the mADC model to compute d' and b_{cc} as described previously. d'_{av} was computed by excluding the lowest angle. Values for the first half and the second half of the data were subtracted from each other. ΔCu and ΔUc_{av} were computed for both halves and were correlated with robust ("percentage-bend") correlations. Corresponding correlation and p-values are shown in (Supplementary Fig.S2). To test whether sensitivity and bias modulations were correlated, we performed a similar split half analysis except that in this case AE(d') and AE(b_{cc}) were computed for each split half of the data, subtracted across the two halves and correlated with each other (Supplementary Fig.S4).

Model comparison analysis. To determine whether the bias quantified by the m-ADC model was a choice bias (shift in choice criteria), or could be explained by sensory input gain alone we compared two models. In the first model (input gain model/Model-δ), input gain (δ; Fig.4E) produced by exogenous cueing varied across locations (Cu, Ip, Co, Op) but the criterion was held uniform across locations. The input gain is modeled as an additive factor to the input (change in Δθ), which increases the gain of the input along the x-axis of the psychophysical function (Fig.4E)[ref.43, main text]. In the second model (choice bias model/Model-c), the input gain was uniform across locations but criteria varied across locations, as in the conventional m-ADC model. In each case, models were compared with the Akaike Information Criterion (AIC) that represents a tradeoff between model complexity (the number of fitted model parameters) and goodness-of-fit (based on the log-likelihood function); a lower AIC score represents a better candidate model. The number of fitted parameters in both of the input gain and choice bias models was 10. We performed these fits using a parameterized form of the m-ADC model using d_{max} (4), θ_{50} (1), criterion (1), δ (4) as parameters for fitting the data with Model-δ, and d_{max} (4), θ_{50} (1), criteria (4), δ (1) in Model-c. AIC values were computed for each trial type and subject separately. Average AIC values with s.e.m error bars are reported in the Results (Fig.4F).

Reaction time regression and prediction analysis. To quantify the effect of sensitivity and bias on reaction times obtained from the behavioral data, we performed a multiple linear regression analysis with AE(RT) as the dependent variable and normalized AE(d') and AE(b_{cc}) as predictors. Each variable was normalized based on z-scoring to make the magnitudes of the regression coefficients, $\beta_{d'}$ and β_{cc}, comparable. To test if the $\beta_{d'}$ and β_{cc} values were significantly different from zero we employed a random permutation test. We created two null distributions for $\beta_{d'}$ and β_{cc} (each) by randomly shuffling subject labels on the predictor variables and recomputing the coefficients over 1000 iterations. The p-values correspond to the proportion of instances in the null distribution which fell below the actual $\beta_{d'}$ and β_{cc} values. We also performed an ANOVA to test for the relative contributions of AE(d') and AE(b_{cc}), in explaining the variance in AE(RT).

In addition, we performed a prediction analysis, by predicting the attentional effect on reaction time for each individual subject using their sensitivity and bias modulations. For this analysis, as before, we fit AE(RT) based on AE(d') and AE(b_{cc}) as predictors using multiple linear regression. The only difference was that this fitting was done by estimating $\beta_{d'}$ and β_{cc} by leaving out one subject at a time. Following this the AE(RT) of the left out subject was linearly estimated based on the AE(d') and AE(b_{cc}) of the left out subject, but using the $\beta_{d'}$ and β_{cc} from the rest of the subjects. We correlated the predicted AE(RT) against the observed AE(RT) with robust correlations ("percentage-bend" correlations). To determine which coefficient – $\beta_{d'}$ or β_{cc} – was more important for RT predictions, we computed confidence intervals for these coefficients based on jack-knife (leave-one-out) standard error estimates[1].

Supplementary Results

Perceptual versus working memory strategies for localizing changes.

Several lines of evidence indicate that subjects were not encoding the grating orientations into working memory on each trial, in our task. First, all four gratings were presented on the screen at all times during the trial except for a very brief (50 ms) blank, thereby precluding the need to store them in working memory. Second, grating orientations were pseudo-randomized on each trial: encoding four arbitrary orientations into working memory on each trial poses a significant challenge, considering that each subject performed up to 480 trials, including training and testing blocks. Finally, if subjects were encoding the gratings into working memory and comparing them with the new set of gratings following reappearance, we would have expected little or no difference between performance in early post-cue (PE) and late post-cue (PL) trials. In other words, subjects could have simply "waited out" to recover from the transient visual interference effect of the cue before making their decision about the change location. Yet, these trends did not occur in our data. Sensitivity (d') was significantly poorer in PE trials than in PL trials at the cued location (PE: d'= 0.40+/-0.04; PL: d'=0.62+/-0.06; p=0.003; Fig. 3B). As a consequence, we observed a strong visual interference effect on d' at the cued location (VE(d') = d'(PE) – d'(PL) = -0.22+/-0.06, p<0.001). This was not an artifact of an urgency signal limiting response times, because no restriction on response times was placed on two-thirds of the subjects (30/45); even in this subset, we observed significant differences in d' between PE and PL trials (VE(d') = -0.28+/-0.06, p<0.001).Taken together, these results suggest that subjects were performing this task as a perceptual change detection task, rather than as a working memory task.

Control experiments: Visual interference effects in pre-cue versus early post-cue conditions.

To test whether the visual interference effects in the pre-cued and the early post-cued trials were comparable, we ran two control experiments.

n=11 subjects (4 females, age range 21-26 yrs), 8 of whom had also participated in the main experiment, participated in the first control experiment. We employed a task design identical with the

exogenous cueing task used in our study, except that subjects were tested on trials with a combination of exogenous and predictive cues. Subjects were tested on two blocks of 60 trials each. The trial structure was identical with that described in the paper, except that we presented a predictive, central arrow cue (0.4 dva; oriented along one of the 4 diagonal directions) for 1500 ms at the onset of each trial along with the fixation cross. On each trial, the arrow cue predicted, with 100% validity, the location of the upcoming exogenous cue. As before one-third of all trials were pre-cue trials (PC) and the remaining two-thirds were equally divided among early and late post-cue trials (PE, PL). Only 25 degree orientation changes were tested, and responses were analyzed, as before, with the m-ADC model.

Many previous studies have shown a strong link between surprise and stimulus-driven attention – exogenous cues are most effective at drawing attention when they are unpredictable in space and time ([2–5] but see [6]).We hypothesized that if the observer had foreknowledge of the location of the exogenous cue before the cue occurred, then, its effect on performance would be related to visual interference and not to exogenous attention. We employed a cue predictive of the upcoming exogenous cue, rather than the upcoming change to avoid performance ceiling effects, and because previous studies have reported dissociable effects of exogenous and endogenous cues on performance[7–9]. If the effects of the pre-cue and early post-cue in this control task were similar, this would provide evidence for comparable visual interference effects of the pre-cue and of the early post-cue.

d's at the predictive (arrow) cued location were numerically higher than those at other uncued locations, in all conditions (d'-PC: Cu=0.67+/-0.13, Uc_{av}=0.54+/-0.11; d'-PE: Cu=0.67+/-0.15; Uc_{av}=0.50+/-0.10; d'-PL: Cu=0.71+/-0.15; Uc_{av}=0.40+/-0.10). An ANOVA revealed a marginally significant effect of location ($d'_{Cu} > d'_{Ucav}$; p=0.06), but not condition (PC, PE, PL; p=0.85). We expect that this increase in d' at the cued location occurred because of the spatially directed arrow cue that preceded the exogenous cue; several previous studies suggest that such overlearned symbols are

effective at orienting attention, in a manner similar to exogenous cues[10–12]. As a result, in this task with spatially predictable exogenous cues, we observed that the difference in d' between the pre-cue and early post-cue trials was statistically indistinguishable from zero (Cu, d': PC-PE=-0.01+/-0.17, p=0.97, signrank test; n=11), as was the difference in (Cu, b_{cc}: PC-PE =-0.04+/-0.08, p=0.99). These results are consistent with the hypothesis that once the attention effects of exogenous cueing were removed by the predictive arrow cue, visual interference effects on d' were statistically indistinguishable between the pre-cue and early post-cue trials.

n=10 subjects (5 females, age range 21-26 yrs), one of whom had also participated in the main experiment, participated in the second control experiment. For this experiment, we employed the following task design: we presented stimuli at only one location on each trial, at predictable times, to minimize spatial and temporal uncertainty associated with the event (change). We expected that these task manipulations would eliminate the "attentional effect" (both spatial and temporal components) of exogenous cueing, such that performance in pre-cue and early post-cue trials was influenced solely by the visual interference effect of the cue.

Stimuli were presented, in turn, at each of the four locations tested in the main experiment; each location was tested for a contiguous block of 30 trials. Each experimental session lasted for 8 blocks for a total of 240 trials per session. In addition, the change was presented, on all trials, at a predictable interval of 2417 ms, following the appearance of the initial fixation cross. One-third of all trials were pre-cue trials (PC), one-third were early post-cue trials (PE) and on the remaining one-third of the trials no cue appeared (NC); the last group of trials provided a control condition with no cueing effects (either attentional or visual interference). For this experiment, we tested only 10 degree orientation changes (to avoid ceiling effects) because we expected the change detection task to be considerably simplified due to eliminating all but one potential stimulus location. Responses were analyzed with a conventional Yes/No SDT model. We tested if psychophysical parameter values were comparable between the pre-cue (PC) and early post-cue (PE) conditions in this control task; the latter would

provide additional evidence for comparable visual interference effects of the pre-cue and of the early post-cue.

We observed that d' for the PC and PE conditions were not significantly different from each other (PC: d'=0.74 +/-0.18; PE: d'=0.67 +/-0.15; p=0.70, signrank test; n=10), as was bias (PC: b_{cc}=-0.01+/-0.06; PE: b_{cc}=-0.08+/-0.04; p=0.28). We also measured the magnitude of the visual interference effect in the PC and PE conditions by subtracting the magnitude of d' and bias from their values in the neutral cueing (NC) condition. Again, these values were not significantly different from each other (PC-NC: d'=0.10 +/- 0.13; PE-NC: d'=0.02 +/-0.14, p=0.70, and PC-NC: b_{cc}=0.09 +/-0.07; PE-NC: b_{cc}=0.02 +/-0.03; p=0.28). The visual interference effect was not significant for any metric, for any condition (p>0.2), possibly because the subject's endogenous attention was already deployed to the location of the (single) stimulus, thereby mitigating the visual interference effect of the exogenous cue.

Taken together, the results of these control experiments indicate that visual interference effects were comparable between pre-cue and early post-cue conditions.

Supporting evidence for attention and visual suppression effects of exogenous cueing.

The global attentional effect, and the local visual suppression effect, of exogenous cueing, were supported by two other lines of evidence. First, a split-half analysis of the data (Supplementary Methods) revealed that modulations of sensitivity due to exogenous attention showed a negative correlation trend across cued and uncued locations (AE(d'): ρ=-0.30, p=0.046; Supplementary Fig.S2), indicating a globally conserved neural resource for sensory processing. In contrast, no such correlation was observed for modulations of the visual interference effect (VE(d'): ρ=-0.13, p=0.389). Second, d' values between the cued and uncued locations were significantly correlated, across subjects, in the PL trials and in PC trials (PL: ρ=0.52; p<0.001, PC: ρ=0.52; p<0.001; Supplementary Fig.S2), but not in the PE trials (PE: ρ=0.25, p=0.97). These results are consistent with the following model: At baseline

(PL trials), average sensitivities were similar across locations for each subject and, therefore, correlated across locations. Pre-cueing (PC trials) did not alter these correlations significantly, because attentional effects of exogenous cueing on d’ operated in a coupled manner (albeit with opposite signs) across cued and uncued locations. In contrast, in PE trials, the visual suppression effect of cueing on d’ were isolated to the cued location, and did not affect d’ at uncued locations, thereby eliminating the correlations across these locations.

Distinguishing between choice bias, sensory input gain and motoric response bias.

To distinguish between choice bias and sensory input bias, we fit two competing models (Supplementary Methods): i) an input gain model (Model-δ), in which the criterion (c) was specified to be the same at all locations while the sensory input gain (δ) was allowed to vary across locations (Fig.4E) and ii) a choice bias model (Model-c), in which the input gain was specified to be the same at all locations while criteria were allowed to vary across locations (Fig.4E). We evaluated the evidence in favor of Model-δ versus Model-c based on the Akaike Information Criterion (AIC), a metric that trades off model complexity against goodness-of-fit; the model with the lowest AIC value is selected as the favored model[13]. The performance of each model is shown in Fig.4F. Comparing the AIC computed by fitting each model to the data revealed that the choice bias model outperformed the input gain model (average AIC, input gain model: 797.1, choice bias model: 790.2, $p<0.001$, signed rank test). We conclude, therefore, that bias modulation due to exogenous cueing was a result of a shift in criterion (choice bias) rather than a change in input bias.

By task design, exogenous cues were not predictive of upcoming changes. Therefore, motoric response biases – for example, those induced by motor preparation for a response to the cued location – are unlikely to have contributed to choice bias. Nevertheless, to distinguish between choice bias and motoric response bias, we availed of the fact that greater motor preparation is likely to produce faster reaction times[14]. Thus, if increased motor bias contributed to increased choice bias at the cued location, we hypothesized that a) the attention effect on RT would be strongly correlated with the

attention effect on b_{cc} at the cued location and b) difference in RT between cued and uncued (Co_{av}) locations would also be correlated with the difference in bias (b_{cc}) between these locations in the PC trials. Neither of these trends occurred in our data (Cu: AE(RT) vs. AE(b_{cc}), $\rho=0.14$, $p=0.375$; RT: Cu-Co_{av} vs. b_{cc}: Cu-Co_{av}, $\rho=0.15$, $p=0.337$). These results indicate that bias modulation by exogenous cueing was not linked to motoric response biases. We also tested whether the correlated modulation of bias (AE(b_{cc})) to the cued and cue-ipsilateral locations (Fig.4D) occurred because of shared motor bias, because responses to changes at both of these locations (Cu, Ip) were made with fingers of the same hand (Supplementary Fig.S1). We hypothesized that if this were the case, we should also observe correlated modulation of reaction times at these locations (AE(RT)). Again, this hypothesis was not true in our data (AE(RT): Cu vs. Ip, $\rho=0.26$, $p=0.108$), indicating that the hemifield specific modulation of bias could not be explained by shared motor bias for responses to these locations.

Testing for reliability of sensitivity and bias modulations.

To ensure that the null effects reported in the text were not due to a lack of reliability of each metric, computed alone, we performed a split half analysis of the data. We selected alternate trials from each block, ensuring equal number of trials for each the angles tested in each half of the data. We estimated attention effects on sensitivity and bias (AE(d') and AE(b_{cc})) by pooling the data across locations and angles tested. We found a strong, and significant correlation in the attention effect on bias (AE(b_{cc}): $r=0.60$, $p<0.001$) and a trend in the correlation for the attention effect on sensitivity (AE(d'): $r=0.28$, $p=0.068$) across the halves. The corresponding values, for the correlation of attention effects for d' versus bias modulation (AE(d') vs. AE(b_{cc})), were not significant (odd trials: $r=0.05$, $p=0.745$; even trials: $r=0.18$, $p=0.232$), in line with results reported in the main text.

Effect of sensitivity and bias on reaction time effects of exogenous cueing.

We fit a multiple linear regression model with AE(RT) as the dependent variable and AE(d') and AE(b_{cc}) as the independent variables; we performed this analysis by normalizing the sensitivity and bias predictors (Supplementary Methods) so that the magnitude of the standardized linear coefficients

β_d and β_{cc} could be directly compared. We found that while β_d was negative and significantly different from zero (β_d=-0.089, $p<0.001$), β_{cc} was positive, but not significantly different from zero (β_{cc}=0.026, $p=0.905$)(Supplementary Fig.S4). Similarly, an ANOVA analysis revealed a main effect of sensitivity (AE(d'): $p=0.003$) on reaction time (AE(RT)), but no main effect of bias (AE(b_{cc}): $p=0.131$); the interaction between sensitivity and bias effects was also not significant ($p=0.345$). Finally, we performed a prediction analysis with a leave-one-out approach: we estimated β_d and β_{cc} on all but one subject in the population, and then predicted the reaction time effect in the left-out subject based on these population β-s and the subject's own sensitivity and bias (Supplementary Methods). This analysis revealed robust prediction accuracies ($\rho=0.42$, $p<0.001$) across the population of subjects. In addition, AE(d') predictors were more important than AE(b_{cc}) predictors for generating successful RT predictions ($p=0.013$, jackknife resampling; Supplementary Methods).

Supplementary References

1. Efron, B. & Stein, C. The Jackknife Estimate of Variance. *Ann. Stat.* **9,** 586–596 (1981).
2. Horstmann, G. The surprise-attention link: a review. *Ann. N. Y. Acad. Sci.* **1339,** 106–115 (2015).
3. Itti, L. & Baldi, P. Bayesian surprise attracts human attention. *Vision Res.* **49,** 1295–1306 (2009).
4. Becker, S. I. & Horstmann, G. Novelty and saliency in attentional capture by unannounced motion singletons. *Acta Psychol. (Amst).* **136,** 290–299 (2011).
5. Schönhammer, J. G. & Kerzel, D. Optimal task-sets override attentional capture by rare cues. *J. Exp. Psychol. Hum. Percept. Perform.* **44,** 681–692 (2018).
6. Meijs, E. L., Klaassen, F. H., Bokeria, L., van Gaal, S. & de Lange, F. P. Cue predictability does not modulate bottom-up attentional capture. *R. Soc. Open Sci.* **5,** 180524 (2018).
7. Bisley, J. W. Neuronal Activity in the Lateral Intraparietal Area and Spatial Attention. *Science (80-.).* **299,** 81–86 (2003).
8. Berger, A., Henik, A. & Rafal, R. Competition between endogenous and exogenous orienting of visual attention. *J. Exp. Psychol. Gen.* **134,** 207–221 (2005).
9. Hopfinger, J. B. & West, V. M. Interactions between endogenous and exogenous attention on cortical visual processing. *Neuroimage* **31,** 774–789 (2006).
10. Reuss, H., Pohl, C., Kiesel, A. & Kunde, W. Follow the sign! Top-down contingent attentional capture of masked arrow cues. *Adv. Cogn. Psychol.* **7,** 82–91 (2011).
11. Eimer, M. Uninformative symbolic cues may bias visual-spatial attention: behavioral and electrophysiological evidence. *Biol. Psychol.* **46,** 67–71 (1997).
12. Hommel, B., Pratt, J., Colzato, L. & Godijn, R. Symbolic Control of Visual Attention. *Psychol. Sci.* **12,** 360–365 (2001).
13. Bozdogan, H. Model selection and Akaike's Information Criterion (AIC): The general theory and its analytical extensions. *Psychometrika* **52,** 345–370 (1987).
14. Stanford, T. R., Shankar, S., Massoglia, D. P., Costello, M. G. & Salinas, E. Perceptual decision making in less than 30 milliseconds. *Nat. Neurosci.* **13,** 379–385 (2010).

Supplementary Figure Legends

Supplementary Figure S1. Model fitting and modulation of responses by exogenous cueing.

A. Response box (RB-840, Cedrus Inc.) showing the configuration for responses for the 4-ADC task shown Fig. 1A (main text).

B. Response proportions fitted with the m-ADC model (ordinate) plotted against observed response proportions (abscissa) for PC (left), PE (middle) and PL (right) trials. Data points: individual 5x5 stimulus-response contingencies for each of the n=45 subjects.(Insets) Distribution of p-values for randomization goodness-of-fit tests.

C. Different response categories (highlighted circles) in the 5x5 stimulus-response contingency table. From left to right: 'Hits' – response indicating the correct location of change; 'False Alarms': response indicating change on no-change trials; 'Correct rejections': no-change response on no-change trials; 'Misses': no-change response on change trials; and 'Mislocalizations': response indicating change at location other than the change location.

D. Correct rejection rates for the three trial types (PC, PE, PL). Other conventions are as in Fig. 2C (main text).

E. Modulation of correct rejection rates with exogenous cueing. Filled bars: Attention effect. Hatched bars: Visual effect. Other conventions are as in Fig. 2D (main text).

F. Reaction times of 'Hits' (averaged across orientation change values) for the four locations (Cu, Ip, Co, Op) and three trial types (PC, PE, PL). Other conventions are as in Fig. 2C (main text).

G. (Top) Modulation of hit-rates by exogenous cueing at cued relative to cue-ipsilateral locations (Cu vs Ip). (Bottom) Same as top panel, but for cued relative to average of cue-contralateral and cue-opposite locations (Cu vs Co_{av}). Filled bars: Attention effect. Hatched bars: Visual effect. Error-bars: s.e.m. across subjects.

H. Same as in panel G, but for false-alarm rates.

I. Same as in panel G, but for reaction times.

Supplementary Figure S2. Effects of exogenous cueing on sensitivity and its modulations.

A. Modulation of sensitivity (averaged across orientation change values) by exogenous cueing for the four locations (Cu, Ip, Co, Op) for full (100%) contrast cues (n=15 subjects). Filled bars: Attention effect. Hatched bars: Visual effect. Other conventions are as in Fig. 2D (main text).

B. Same as in panel A but for half (50%) contrast cues (n=30 subjects).

C. (Top) Covariation of attentional modulation of sensitivity (AE(d')) by exogenous cueing at cued location versus at uncued locations based on split half analysis of the data (see text for details). Circles: Data for each subject (n=45). Line: best linear fit. ρ and p values for each correlation are indicated in the upper right corner of each sub-panel. (Bottom) Same as top panel, but for visual effect of exogenous cueing (VE(d')).

D. Difference of d' values at the cued location relative to uncued locations (average across Ip, Co, Op) for the three trial types (PC, PE, PL). Other conventions are as in Fig.2C (main text).

E. Covariation of sensitivity (d') values at cued location with uncued locations for PC (left), PE (middle), and PL (right) trials. ρ and p values for each correlation are indicated in the upper right corner of each sub-panel.

F. Sensitivity (d') (data pooled across n=45 subjects and averaged across orientation change values) for the four locations (Cu, Ip, Co, Op) and three trial types PC (left), PE (middle), and PL (right) trials. Other conventions are as in Fig. 2C (main text).

Supplementary Figure S3. Effects of exogenous cueing on bias and its modulations.

A. Difference of bias (b_{cc}) values at the cued location relative to Ip location (left) and average of contralateral locations (Co, Op) (right) for the three trial types (PC, PE, PL). Other conventions are as in Fig.2C (main text).

B. Covariation of difference of bias (b_{cc}) across the cued and cue-ipsilateral location (Cu-Ip) versus the difference of values across cued and both contralateral location (Cu-Co), for PE (left) and PL (right) trials. Triangles: Data for individual subjects (n=45). ρ and p values for each correlation are indicated in the upper right corner of each sub-panel; line: best linear fit.

C. Choice bias (b_{cc}) (data pooled across n=45 subjects and averaged across orientation change values) for the four locations (Cu, Ip, Co, Op) and three trial types PC (left), PE (middle), and PL (right) trials. Other conventions are as in Fig. 2C (main text).

Supplementary Figure S4. Effect of exogenous cueing on sensitivity, bias and reaction times and their modulations.

A. Covariation between attentional modulations of sensitivity (AE(d')) and bias (AE(b_{cc})) across blocks at each of the four locations, obtained by computing AE(d') or AE(b_{cc}) for each half of the data, and subtracting the corresponding quantities across the two halves. Data points: Individual subjects (n=45). ρ and p values for each correlation are indicated in the upper right corner of each sub-panel. Other conventions are as in Fig.5B (main text).

B. Regression coefficients for AE(d') (β_d) and AE(b_{cc}) (β_{cc}) for predicting the attentional modulation of reaction time (AE(RT)). Error bars indicate jack-knife s.e.m. Asterisks: significance levels (***-$p<0.001$).

Supplementary Figure S1

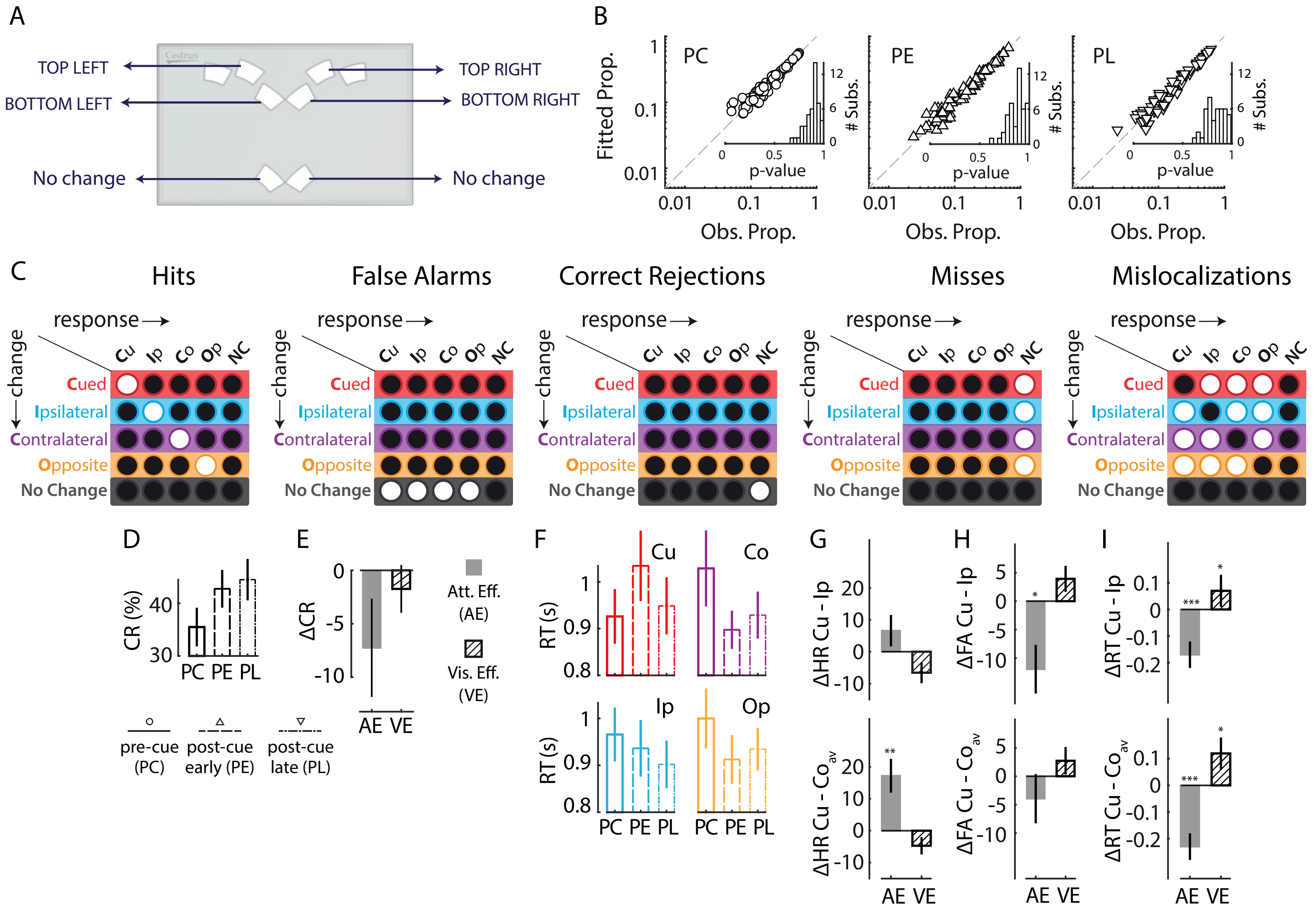

Supplementary Figure S2

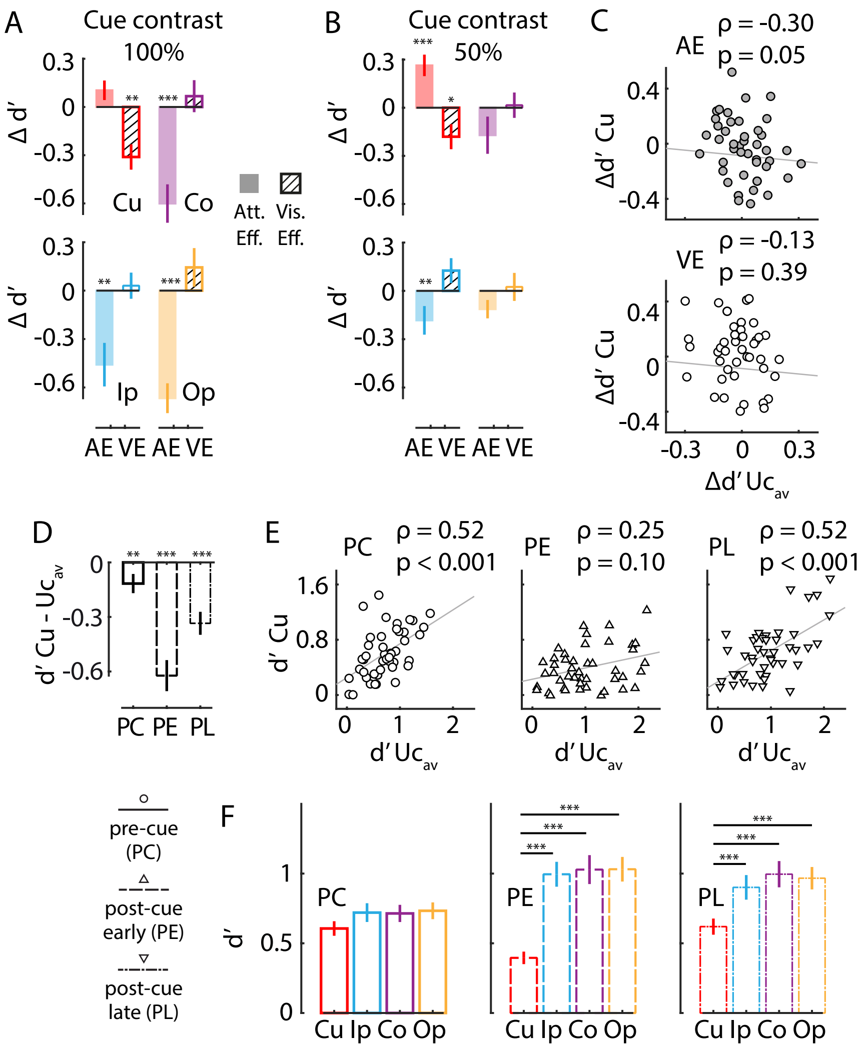

Supplementary Figure S3

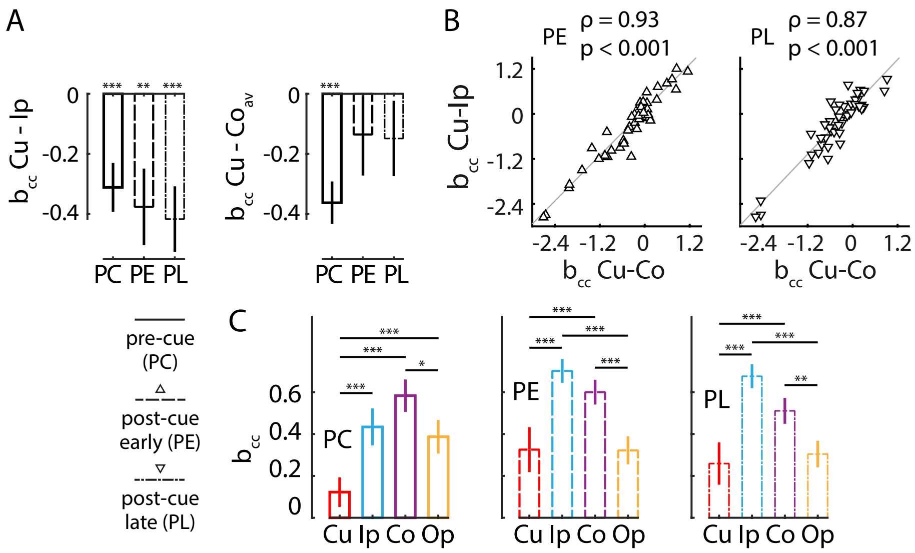

Supplementary Figure S4

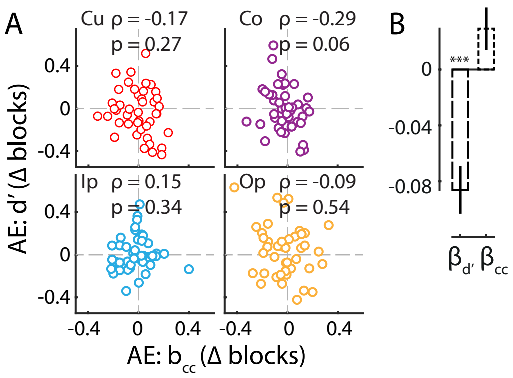

Supplement: Supplementary file 1 — Supplementary Information [file 41598_2019_42759_MOESM1_ESM.pdf]
